# Optical Isolator Utilizing Surface Plasmons

**DOI:** 10.3390/ma5050857

**Published:** 2012-05-16

**Authors:** Vadym Zayets, Hidekazu Saito, Koji Ando, Shinji Yuasa

**Affiliations:** Spintronic Research Center, National Institute of Advanced Industrial Science and Technology (AIST), Umezono 1-1-1, Tsukuba, Ibaraki, 305-8568, Japan; E-Mails: h-saitoh@aist.go.jp (H.S.); ando-koji@aist.go.jp (K.A.); yuasa-s@aist.go.jp (S.Y.)

**Keywords:** optical isolator, surface plasmons, magneto-optical effect

## Abstract

Feasibility of usage of surface plasmons in a new design of an integrated optical isolator has been studied. In the case of surface plasmons propagating at a boundary between a transition metal and a double-layer dielectric, there is a significant difference of optical loss for surface plasmons propagating in opposite directions. Utilizing this structure, it is feasible to fabricate a competitive plasmonic isolator, which benefits from a broad wavelength operational bandwidth and a good technological compatibility for integration into the Photonic Integrated Circuits (PIC). The linear dispersion relation was derived for plasmons propagating in a multilayer magneto-optical slab.

## 1. Introduction

The magneto-optical (MO) effect is important for a variety of applications. A unique feature of the MO effect is non-reciprocity. The optical properties of non-reciprocal devices are different for two opposite directions of light propagation. The non-reciprocal effect can occur only in a MO material and the important optical non-reciprocal devices, such as an optical isolator and an optical circulator, can only be fabricated by utilizing MO materials. An optical isolator is an important component of optical communications systems. It protects optical elements from back-reflected light. The protection by the optical isolator is essential for the normal operation of optical components. For example, the operation of high-speed optical amplifiers and high-speed laser diodes is impossible without protection by an optical isolator, because of the gain modulation by back-traveling light.

The performance of an optical isolator is characterized by 3 main parameters: the isolation ratio, the insertion loss and the operational wavelength bandwidth. At present, the bulk-type optical isolator is commercially available. The structure of the bulk-type optical isolator is rather simple. It consists of a magnetic garnet inserted between two polarizers. Since both magnetic garnets with low absorption and the polarizers with low insertion loss and high extinction ratio are available, the performance of the present commercial optical isolator is close to perfect. The optical bulk isolators with isolation ratio of 40 dB, insertion loss of 0.7 dB and bandwidth of 20 nm are commercially available.

The integration of optical elements into Photonic Integrated Circuits (PIC) is an important task, because it may reduce cost and improve performance of high-speed optical data processing circuits for the high-speed optical networks. The integration of an optical isolator is important for PIC, because the problem of back-reflected light is more severe in the case of integrated optical elements. However, the optical isolator is one of the few optical components, which has not yet been integrated into commercial PIC. At present, there is a strong industrial demand for the integrated optical isolator.

It is difficult to integrate the optical isolator into PIC for the following reason. The indispensable component of any optical isolator is a magneto-optical (MO) material. Traditionally, the magnetic garnets are used as a transparent MO material. However, it is difficult to grow the high-crystal-quality magnetic garnets on semiconductors substrates such as Si, GaAs, InP, which are used as a substrate for PIC, because the conventional growth temperature of the garnets is above the melting temperature of these substrates.

To overcome this problem several solutions have been proposed. A wafer bonding between garnets and semiconductors [1] and sputtering of garnet on Si [2] were successfully used to fabricate the isolator on a Si substrate. Also, diluted magnetic semiconductors CdMnTe and CdMnHgTe [3] have been used for the fabrication of optical isolator on a GaAs substrate. Even though a good performance of the above-mentioned standalone isolators fabricated on the semiconductor substrates were achieved, the integration of the isolators with other optical elements on the same substrate has not been demonstrated yet. The integration is difficult, because MO and optical properties of the garnets and diluted magnetic semiconductors (DMS) significantly depend on their crystal quality. As the crystal quality of garnets or DMS degrades, the MO constants in these materials are reduced and the optical loss is increased. Even if it is possible to keep a high crystal quality of the MO material during the fabrication of a standalone isolator, the crystal quality usually degrades during the fabrication of other elements of PIC. That is the reason why there are still difficulties to integrate a garnet-based isolator or a DMS-based isolator into a commercial PIC.

Ferromagnetic metals are attractive as an MO material for the optical isolator. They have very large MO constants and importantly they are technologically compatible with the fabrication technology of PIC. For the ferromagnetic metals the conventional sputtering and lift-off techniques may be used. They do not degrade during the fabrication of other optical components of PIC. There is one significant demerit of the ferromagnetic metals. They significantly absorb light. To overcome this problem, the design of an isolator, in which the optical absorption by a ferromagnetic metal is compensated by an optical gain of a semiconductor optical amplifier, has been proposed [4] and realized [5,6,7]. This type of isolator provides high isolation and has low optical insertion loss. However, the required operational pump current for this isolator is very high. At present, it is about 100 mA. The required current is unacceptably high for PIC. That is a reason why this type of isolator has not been integrated into PIC as well.

Here we propose to utilize the surface plasmons propagating at the surface of the ferromagnetic metals to fabricate an efficient optical isolator.

It should be noticed that since the performance of the bulk isolator is nearly excellent, at present it is unfeasible that an isolator integrated into PIC would reach a similar performance. For the merit of the integration, some penalties should be paid. Therefore, some parameters of the integrated isolator should be compromised. For example, in the case when the operational wavelength bandwidth may be compromised, a ring-type isolator may be a good option [1,2]. The ring-type isolator has very narrow bandwidth of about 0.01 nm.

The surface plasmons intrinsically have a substantial optical loss. It is unlikely that the insertion loss and the isolation ratio of the plasmonic isolator would be the same as those of the bulk isolator. However, the ferromagnetic metals have very low wavelength dispersion of the optical and MO constants and the plasmonic isolator might be a good option for the integrated isolator when a wide operational wavelength bandwidth is required. As was mentioned above, the technological compatibility with PIC fabrication technology is another important merit of the plasmonic isolator.

MO effect for the surface plasmons was studied both theoretically and experimentally [8,9,10,11]. Here we study how to optimize the MO effect for the surface plasmons in order to fabricate an efficient optical isolator. For the optical isolator we are proposing to use single-surface plasmons propagating along a double-dielectric/ferromagnetic-metal interface. We have already shown [12] that it is possible to achieve a significant reduction of the optical loss and the enhancement of MO effect for surface plasmons propagating in this structure. We discuss MO properties of surface plasmons in the case when a magnetic field is applied perpendicularly to the plasmon propagation direction and in the film plane. In this case the unique properties of the transverse non-reciprocal MO (nMO) effect are utilized for MO enhancement [12]. The transverse nMO effect occurs in the case when light propagates perpendicularly to the magnetic field. In reference [12] we have shown that light can experience the transverse nMO effect only when it propagates in the vicinity of a boundary between two materials and the optical field at least in one material is evanescent. The transverse nMO effect occurs because the polarization of light, which has an evanescent component, rotates around an axis, which is parallel to the magnetic field and perpendicular to the light propagation direction. The magnitude of the transverse nMO effect is comparable to or often greater than the magnitude of the conventional longitudinal nMO effect. In contrast to the longitudinal nMO effect, the transverse nMO effect can be significantly magnified by optimizing the device structure.

A linear dispersion relation, which describes optical and MO properties of the surface plasmons propagating in single-dielectric, double-dielectric and multilayer structures, will be derived in Section 4.

## 2. Surface Plasmons

Surface plasmons have proved to be very efficient in localizing and guiding photonic signal on a scale which is hardly reachable with conventional dielectric waveguides. This makes them a prospective candidate for downsizing integrated optical circuits. A surface plasmon is an electromagnetic wave coherently coupled to electron oscillations and propagating in a wave-like fashion along a metal-dielectric interface. Currently, two types of the surface plasmons are utilized in optical applications [13]. A long-range surface plasmon propagates in a thin metallic film. A single-surface plasmon propagates along a boundary between a relatively-thick metallic film and a dielectric. Only the single-surface plasmons will be discussed in this paper.

**Figure 1:**
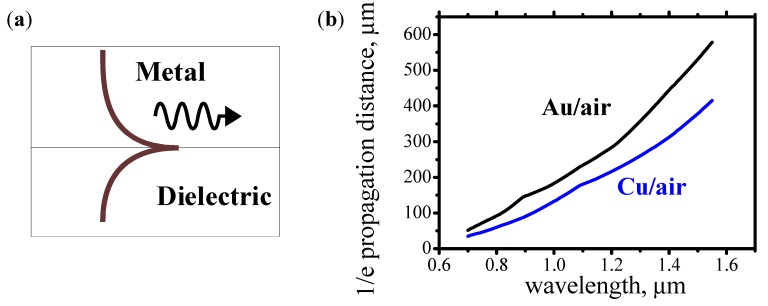
(**a**) Schematic diagram showing the intensity of optical field for a surface plasmon propagating along the metal-dielectric interface. Brown line is intensity distribution across the interface; (**b**) 1/e propagation distance for the surface plasmons propagating along Au/air and Cu/air interfaces.

Figure 1a shows the optical intensity distribution for a plasmon propagating along metal-dielectric interface. Since light is tightly confined near the metal-dielectric interface, the plasmons are often used for the dense integration of optical elements or when an ultra dense focusing of light is required (for example, MO recording or medical applications).

For the surface plasmons, optical field is distributed partially inside the metal and partially inside the dielectric. Since the metal absorbs light, the plasmons always experience optical loss. However, the plasmon’s optical loss is significantly smaller than the optical loss for light, which is propagating through a bulk metal. This is due to the fact that in the case of plasmons a substantial amount of light energy propagates inside the dielectric and only a tiny amount propagates inside the skin depth in the metal. Particularly, the optical loss is small for plasmons in structures, which contain a low-resistance metal like Au, Cu, Al, Ag. Figure 1b shows 1/e propagation distance of plasmons in low-resistance metals Au and Cu. For these metals, the plasmon’s 1/e propagation distance is long, about 200–500 μm. Because of low optical loss, mainly these metals are used for the variety of plasmonic applications. However, Au and Cu are not ferromagnetic metals and a ferromagnetic metal is required for the fabrication of an optical isolator.

**Figure 2:**
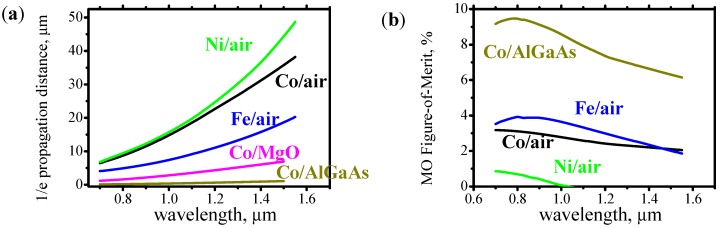
(**a**) 1/e propagation distance, and (**b**) magneto-optical Figure-of-Merit for optical plasmons propagating along Ni/air, Co/air, Fe/air, Co/MgO and Co/Al_0.5_Ga_0.5_As interfaces.

Figure 2a shows 1/e plasmon’s propagation distance in the ferromagnetic metals Fe, Ni, Co. Since the resistance of these metals is higher, the plasmon’s optical loss is also higher and the 1/e propagation distance is significantly shorter than in the case of the surface plasmons in Au and Cu. However, the problem of the high optical loss could be resolved by shortening device length. Since MO constants of the ferromagnetic metals are very large, even for a short propagation distance the plasmons might provide a high isolation and a sufficiently low insertion loss. The MO Figure-of-Merit (FoM), is a ratio of optical isolation to insertion loss.

(1) $$FoM=\frac{isolation}{loss}=\frac{loss_{forward}-loss_{backward}}{\left(loss_{forward}+loss_{backward}\right)/2}$$

MO FoM is a value, which numerically estimates the ability of the plasmons to provide the high isolation at required low insertion loss.

Figure 2b shows the MO FoM for plasmons propagating along Fe/air, Co/air, Ni/air, Co/AlGaAs interfaces. In all cases the FoM is about 2–8%. The MO FoM for the plasmons is comparable to MO FoM for a waveguide mode in optical waveguide covered by a ferromagnetic metal. As was discussed above this value might not be sufficient to fabricate an efficient plasmonic isolator. The data were calculated by solving Maxwell’s equations (Section 4) and utilizing known optical and magneto-optical constants of metals [15,16,17] and semiconductors [18,19].

In the next section we will show that it is possible to reduce the optical loss and to increase the MO FoM in plasmonic waveguides using a dielectric, which consists of two materials with substantially different refractive indexes.

**Figure 3:**
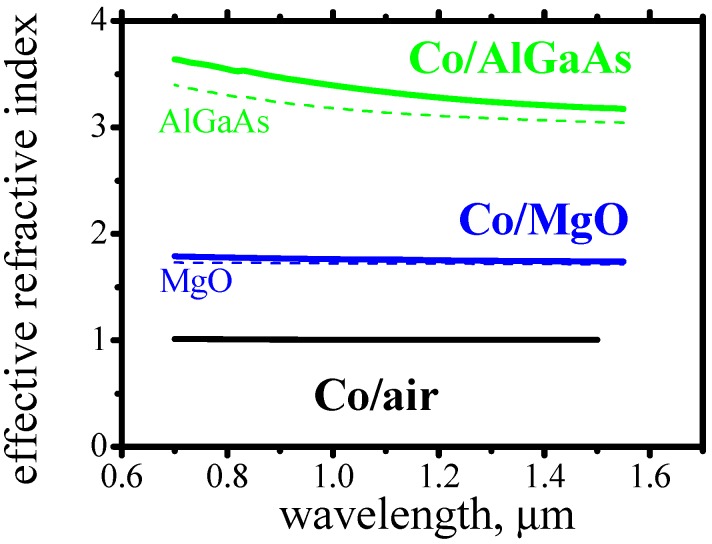
Effective refractive indexes of surface plasmons propagating along Co/Al_0.5_Ga_0.5_As, Co/MgO and Co/air interfaces (solid lines) and refractive index of Al_0.5_Ga_0.5_As and MgO (dashed lines).

It is worth to mention two important facts about the surface plasmons. As can be noticed from Figure 2a, the optical loss of surface plasmons depends significantly on the refractive index of the dielectric. The higher the refractive index of the dielectric, the larger loss the plasmons experience. The second fact is that the effective refractive index [20] of surface plasmons is always very close to the refractive index of the dielectric. Figure 3 shows the effective index of surface plasmons propagating along Co/AlGaAs interface. The dashed lines show the refractive index of the corresponding dielectric. In all cases the plasmon’s effective refractive index is only slightly larger than the refractive index of the dielectric.

## 3. Enhancement of MO Effect in Double-Layer-Dielectric Plasmonic Waveguide

**Figure 4:**
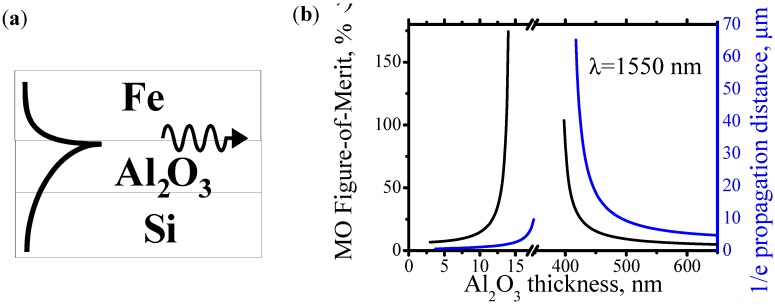
(**a**) Structure of Fe/Al_2_O_3_/Si plasmonic waveguide with a double-layer dielectric; (**b**) Magneto-optical Figure-of-Merit and 1/e propagation distance for plasmons in Fe/Al_2_O_3_/Si plasmonic waveguide.

Figure 4a shows an example of the plasmonic waveguide with a double-layer dielectric. It consists of Fe as a ferromagnetic metal, Al_2_O_3_ as a dielectric of low refractive index and Si as a dielectric of high refractive index. Figure 4b shows calculated plasmon’s 1/e propagation distance and MO FoM in this structure as a function of Al_2_O_3_ thickness. The structure does not support the plasmons when Al_2_O_3_ thickness is between 15 nm and 395 nm. Near “cutoff” thicknesses of 15 nm and 395 nm, the MO FoM significantly increases and the plasmon’s propagation distance becomes longer.

The existence of the cutoff thicknesses can be understood as follows. As was explained above, the effective refractive index of the plasmons is always very close to the refractive index of the dielectric. It is an important condition ensuring the plasmon confinement near the interface. In case of the double-layer dielectric, which consists of Al_2_O_3_ and Si, the plasmon’s effective index is close to the refractive index of Si when Al_2_O_3_ is very thin and it is close to the refractive index of Al_2_O_3_ when Al_2_O_3_ is very thick. Since the difference between refractive indexes of Al_2_O_3_ and Si is large, for the intermediate thicknesses of Al_2_O_3_ the condition of the plasmon confinement near the interface could not be satisfied.

The reason for the reduction of plasmon’s optical loss near the cutoff is the following: When Al_2_O_3_ thickness approaches a “cutoff’ thickness, the penetration depth of the plasmon’s optical field into silicon significantly increases, but the penetration depth into the iron remains unchanged. That means that the amount of the optical field decreases inside the metal and increases inside the silicon. Therefore, the optical field is “pushed out” from the iron. This causes a significant decrease of optical loss and increase of the plasmon’s propagation distance. The following explains the increase of MO FoM near the cutoff thickness. A change of the refractive index of the metal changes the value of the “cutoff’ thickness. The plasmon’s optical loss sharply decreases when the Al_2_O_3_ thickness approaches the “cutoff’ thickness. Therefore, in the case of Al_2_O_3_ thickness close to the “cutoff’ thickness, a small MO change of the refractivity of the metal causes a significant MO change of the plasmon’s optical loss.

As seen from Figure 4b, the MO FoM may reach 100%. This is a significant enlargement of MO FoM compared to 2–8% of the MO FoM, which is the most that can be achieved for the structure in which only one material of the dielectric is used (Figure 2b).

**Figure 5:**
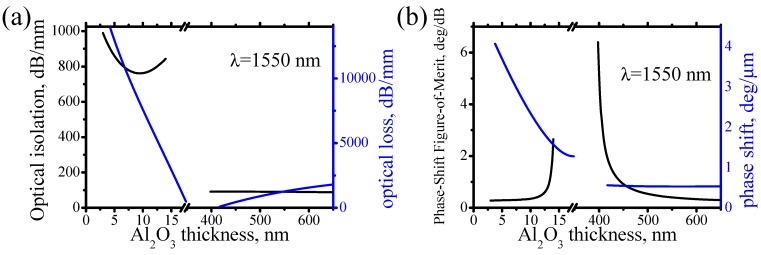
(**a**) Optical isolation and loss, and (**b**) non-reciprocal phase shift and Figure-of-Merit for the non-reciprocal phase shift for the surface plasmons in a Fe/Al_2_O_3_/Si plasmonic waveguide.

Figure 5a compares optical isolation and optical loss in Fe/Al_2_O_3_/Si plasmonic waveguide with a double-layer dielectric. When Al_2_O_3_ thickness approaches the cutoff thicknesses of 16 nm and 400 nm, the optical isolation increases and the optical loss decreases. It should be noted that the optical isolation and loss is significantly larger near the cutoff thickness of 16 nm than near the cutoff thickness of 400 nm, even though MO FoM near both cutoff thicknesses is comparable (see Figure 4b). Therefore, depending on the application, either a longer plasmonic isolator or a shorter plasmonic isolator can be made. For the longer plasmonic isolator, an Al_2_O_3_ thickness of a little more than 400 nm should be used and for the shorter plasmonic isolator an Al_2_O_3_ thickness a little less than 16 nm should be used.

As was shown above, there is a significant difference of optical loss for the surface plasmons propagating in a double-layer-dielectric plasmonic waveguide. It should be noticed that in a MO plasmonic waveguide it is not only the plasmon’s absorption coefficient, which is dependent on a plasmon’s propagation direction, but the effective refractive index depends on propagation direction as well. Therefore, a phase shift, which the plasmons experience after propagation through the MO plasmonic waveguide, is different for two opposite propagation directions. The non-reciprocal phase shift for waveguide modes in dielectric MO waveguide was used to fabricate an efficient MO isolator [1,2,14]. It is interesting to examine how large the non-reciprocal phase shift is for the plasmon and whether there is any enhancement of the non-reciprocal phase shift in the case of a double-dielectric plasmonic waveguide. Since the optical loss of the plasmons is not negligible, the non-reciprocal phase shift per given optical loss is important value for practical application. Therefore, another Figure-of-Merit for the non-reciprocal phase shift can be defined as a ratio of the non-reciprocal phase shift to the optical loss:

$$FoM\_PhaseShift=\frac{Phase_{forward}-Phase_{backward}}{\left(loss_{forward}+loss_{backward}\right)/2}$$

Figure 5b shows non-reciprocal phase shift and Figure-of-Merit for the non-reciprocal phase shift in Fe/Al_2_O_3_/Si plasmonic waveguide with a double-layer dielectric. There is no enhancement of the non-reciprocal phase shift, but there is an enhancement of FoM for the non-reciprocal phase shift near the cutoff thicknesses, because of the loss reduction. Even though the enhancement of FoM for the non-reciprocal phase shift is smaller than the enhancement of FoM for the non-reciprocal loss, it is still prominent.

## 4. Dispersion Relation for Plasmons Propagating in Multi-Layer MO Slab

In the following we will derive the dispersion relation, which describes the MO effect for the surface plasmons propagating in a multilayer MO slab.

**Figure 6:**
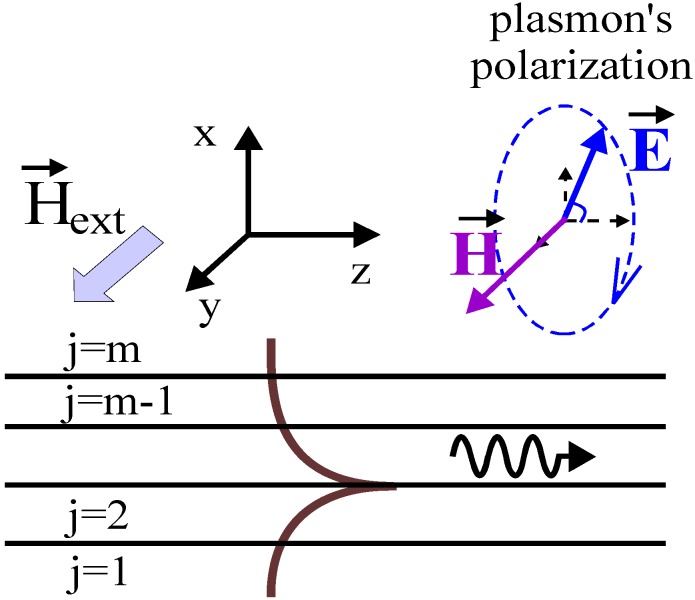
Schematic diagram showing the intensity of optical field for a surface plasmon propagating in plasmonic waveguide, which consists of m-layers. Plasmon’s propagation direction is along the z-axis. External magnetic field is applied along the y-axis. For plasmon’s polarization the magnetic field is directed along the y-axis and the electric field rotates in the xz-plane around the y-axis. Because of the polarization rotation around the direction of the external magnetic field, the surface plasmons experience a substantial transverse non-reciprocal MO effect.

The plasmon’s propagation direction is along the z-axis, the surface normal is along the x-direction and the magnetic field is applied along the y-direction (Figure 6). For this direction of the magnetic field, the permittivity tensor for the ferromagnetic metal will be

(2) $$\hat{\varepsilon }=(\begin{array}{l} \;\varepsilon_{d}\;\;\;\;\;\;0\;\;\;\;-i\cdot \gamma \\ \;\;0\;\;\;\;\;\;\varepsilon_{d}\;\;\;\;\;\;0\;\;\\ i\cdot \gamma \;\;\;\;\;0\;\;\;\;\;\;\varepsilon_{d}\; \end{array})$$

where both $\varepsilon_{d}$ and γ have non-zero real and imaginary parts.

Let’s consider a multilayer MO slab, which consist of m layers and ${\hat{\varepsilon }}_{j}$ and t_j_ the permittivity tensor (2) and the thickness of each j-layer, respectively. For a plain wave

(3) $$E_{x}^{j},E_{z}^{j},H_{y}^{j}\sim e^{\frac{2\pi }{\lambda }i\left(-c\cdot t+k_{xj}x+k_{z}z\right)}$$

a solution for Maxwell’s equations

(4) $$\begin{array}{cc} rot({\vec{H}}_{j})=\frac{1}{c}\frac{\partial }{\partial t}\left({\hat{\varepsilon }}_{j}\cdot {\vec{E}}_{j}\right); & rot({\vec{E}}_{j})=-\frac{1}{c}\frac{\partial }{\partial t}\left({\vec{H}}_{j}\right) \end{array}$$

will be $k_{xj}^{2}=\varepsilon_{dj}-k_{z}^{2}-\gamma_{j}^{2}/\varepsilon_{dj}$

(5) $$\left[\begin{array}{c} E_{x}^{j}\\ H_{y}^{j} \end{array}\right]=\left[\begin{array}{c} \frac{i\cdot \gamma_{j}-k_{z}k_{xj}}{\varepsilon_{dj}-k_{z}^{2}}\\ \frac{i\cdot \gamma_{j}\cdot k_{z}-\varepsilon_{dj}\cdot k_{xj}}{\varepsilon_{dj}-k_{z}^{2}} \end{array}\right]E_{z}^{j}$$

The optical field in j-layer can be described as

(6) $$\left[\begin{array}{c} E_{z}^{j}\\ H_{y}^{j} \end{array}\right]=\left\{A_{f}^{j}e^{i\frac{2\pi }{\lambda }k_{xj}(x-x_{j})}\left[\begin{array}{c} 1\\ \frac{i\cdot \gamma_{j}\cdot k_{z}-\varepsilon_{dj}\cdot k_{xj}}{\varepsilon_{dj}-k_{z}^{2}} \end{array}\right]+A_{b}^{j}e^{-i\frac{2\pi }{\lambda }k_{xj}\left(x-x_{j}\right)}\left[\begin{array}{c} 1\\ \frac{i\cdot \gamma_{j}\cdot k_{z}+\varepsilon_{dj}\cdot k_{xj}}{\varepsilon_{dj}-k_{z}^{2}} \end{array}\right]\right\}e^{\frac{2\pi }{\lambda }i\left(-c\cdot t+k_{z}z\right)}+c.c.$$

where $A_{b}^{j}$ and $A_{f}^{j}$ are unknowns, x_j_ is the coordinate of boundary between j and (j+1) layers and c.c. is complex conjugative. Introducing new unknowns

(7) $$\begin{array}{cc} Z_{j}=\frac{A_{f}^{j}-A_{b}^{j}}{A_{f}^{j}+A_{b}^{j}} & B_{j}= \end{array}A_{f}^{j}+A_{b}^{j}$$

the Equation (7) will be

(8) $$\left[\begin{array}{c} E_{z}^{j}\\ H_{y}^{j} \end{array}\right]=2\cdot B_{j}\left\{\text{cos}\left(\frac{2\pi }{\lambda }k_{xj}(x-x_{j})\right)\left[\begin{array}{c} 1\\ \frac{i\cdot \gamma_{j}\cdot k_{z}-Z_{j}\cdot \varepsilon_{dj}\cdot k_{xj}}{\varepsilon_{dj}-k_{z}^{2}} \end{array}\right]+i\cdot \text{sin}\left(\frac{2\pi }{\lambda }k_{xj}(x-x_{j})\right)\left[\begin{array}{c} Z_{j}\\ \frac{i\cdot \gamma_{j}\cdot k_{z}\cdot Z_{j}-\varepsilon_{dj}\cdot k_{xj}}{\varepsilon_{dj}-k_{z}^{2}} \end{array}\right]\right\}e^{\frac{2\pi }{\lambda }i\left(-c\cdot t+k_{z}z\right)}+c.c.$$

Applying boundary conditions $\begin{array}{cc} E_{z}=const & H_{y}=const \end{array}$ at boundary between j and j+1 layers, we have

(9) $$\begin{array}{l} B_{j}\left\{\text{cos}\left(\frac{2\pi }{\lambda }k_{xj}\left(x_{j+1}-x_{j}\right)\right)\left[\begin{array}{c} 1\\ \frac{i\cdot \gamma_{j}\cdot k_{z}-Z_{j}\cdot \varepsilon_{dj}\cdot k_{xj}}{\varepsilon_{dj}-k_{z}^{2}} \end{array}\right]+i\cdot \text{sin}\left(\frac{2\pi }{\lambda }k_{xj}\left(x_{j+1}-x_{j}\right)\right)\left[\begin{array}{c} Z_{j}\\ \frac{i\cdot \gamma_{j}\cdot k_{z}\cdot Z_{j}-\varepsilon_{dj}\cdot k_{xj}}{\varepsilon_{dj}-k_{z}^{2}} \end{array}\right]\right\}=\\ =B_{j+1}\left[\begin{array}{c} 1\\ \frac{i\cdot \gamma_{j+1}\cdot k_{z}-Z_{j+1}\cdot \varepsilon_{d_{\left(j+1\right)}}\cdot k_{x(j+1)}}{\varepsilon_{d(j+1)}-k_{z}^{2}} \end{array}\right] \end{array}$$

Simplifying Equation (9) we obtain

(10) $$\begin{array}{l} B_{j+1}=B_{j}\left(\text{cos}\left(\frac{2\pi }{\lambda }k_{xj}t_{j}\right)+i\cdot Z_{j}\cdot \text{sin}\left(\frac{2\pi }{\lambda }k_{xj}t_{j}\right)\right)\\ \frac{i\cdot \gamma_{j+1}\cdot k_{z}-Z_{j+1}\cdot \varepsilon_{d(j+1)}\cdot k_{x(j+1)}}{\varepsilon_{d(j+1)}-k_{z}^{2}}=\frac{i\cdot \gamma_{j}\cdot k_{z}}{\varepsilon_{dj}-k_{z}^{2}}+\frac{-\varepsilon_{dj}\cdot k_{xj}}{\varepsilon_{dj}-k_{z}^{2}}\frac{Z_{j}\cdot \text{cos}\left(\frac{2\pi }{\lambda }k_{xj}t_{j}\right)+i\cdot \text{sin}\left(\frac{2\pi }{\lambda }k_{xj}t_{j}\right)}{\text{cos}\left(\frac{2\pi }{\lambda }k_{xj}t_{j}\right)+i\cdot Z_{j}\cdot \text{sin}\left(\frac{2\pi }{\lambda }k_{xj}t_{j}\right)} \end{array}$$

From the second equation, the value of Z_j+1_ can be found when value of Z_j_ is known as

(11) $$Z_{j+1}\cdot \varepsilon_{d_{\left(j+1\right)}}\cdot k_{x(j+1)}=i\cdot \gamma_{j+1}\cdot k_{z}-\frac{\varepsilon_{d(j+1)}-k_{z}^{2}}{\varepsilon_{dj}-k_{z}^{2}}\left[i\cdot \gamma_{j}\cdot k_{z}-\varepsilon_{dj}\cdot k_{xj}\frac{Z_{j}\cdot \text{cos}\left(\frac{2\pi }{\lambda }k_{xj}t_{j}\right)+i\cdot \text{sin}\left(\frac{2\pi }{\lambda }k_{xj}t_{j}\right)}{\text{cos}\left(\frac{2\pi }{\lambda }k_{xj}t_{j}\right)+i\cdot Z_{j}\cdot \text{sin}\left(\frac{2\pi }{\lambda }k_{xj}t_{j}\right)}\right]$$

The Equation (11) in a compact form will be

(12) $$Z_{j+1}=F_{j+1,j}\left[T\prime_{j}\left[Z_{j}\right]\right]$$

where

(13) $$\begin{array}{l} T\prime_{j}\left[Z\right]=\frac{Z+i\cdot tan\left(\frac{2\pi }{\lambda }k_{xj}t_{j}\right)}{1+i\cdot Z\cdot tan\left(\frac{2\pi }{\lambda }k_{xj}t_{j}\right)}\\ F_{j+1,j}\left[Z\right]=\left[i\cdot \gamma_{j+1}\cdot k_{z}-\left(i\cdot \gamma_{j}\cdot k_{z}-Z\cdot \varepsilon_{dj(j)}\cdot k_{xj}\right)\frac{\varepsilon_{d(j+1)}-k_{z}^{2}}{\varepsilon_{dj}-k_{z}^{2}}\right]/\left(\varepsilon_{d(j+1)}\cdot k_{x(j+1)}\right) \end{array}$$

Knowing the value of Z_1_ the value of Z_2_ can be calculated from Equation (13). Knowing the value of Z_2_ the value of Z_3_ can be calculated from Equation (13) and so on.

(14) $$Z_{3}=F_{3,2}\left[T\prime_{2}\left[Z_{2}\right]\right]=F_{3,2}\left[T\prime_{2}\left[F_{2,1}\left[T\prime_{1}\left[Z_{1}\right]\right]\right]\right]$$

Therefore, the values of Z for all layers can be calculated from Equation (12), if Z of one layer is known. T’_j_ describes the variation of Z across j-layer, F_j+1,j_ describes of change Z across the boundary between j and j+1 layers. Noticing that

(15) $$\begin{array}{cc} F_{j+1,j}\left[F_{j,j+1}\left[Z\right]\right]=1 & T_{j}\left[T\prime_{j}\left[Z\right]\right] \end{array}=1$$

where

(16) $$T_{j}\left[Z\right]=\frac{Z-i\cdot tan\left(\frac{2\pi }{\lambda }k_{xj}t_{j}\right)}{1-i\cdot Z\cdot tan\left(\frac{2\pi }{\lambda }k_{xj}t_{j}\right)}$$

The Equation (12) can be solved for Z_j_ as

(17) $$Z_{j}=T_{j}\left[F_{j,j+1}\left[Z_{j+1}\right]\right]$$

To derive plasmon’s dispersion relation, we use the fact that the value of Z is known for the top and the bottom layers. In the case of a surface plasmon, light is confined near the metal/dielectric interface, therefore $\left|\vec{E}\right|\to 0$ when x→±∞. For the bottom layer number 1, which is infinite when x→−∞ , Equation (6) will be

(18) $$\left[\begin{array}{c} E_{z}^{1}\\ H_{y}^{1} \end{array}\right]=\left\{A_{b}^{1}e^{-i\frac{2\pi }{\lambda }k_{x1}\left(x-x_{1}\right)}\left[\begin{array}{c} 1\\ \frac{i\cdot \gamma_{1}\cdot k_{z}+\varepsilon_{d1}\cdot k_{x1}}{\varepsilon_{d1}-k_{z}^{2}} \end{array}\right]\right\}e^{\frac{2\pi }{\lambda }i\left(-c\cdot t+k_{z}z\right)}+c.c.$$

where Im(kx1)>0. From Equation (7) we have

(19) $$Z_{1}=-1$$

For the top layer number m, which is infinite when x→∞, Equation (6) will be

(20) $$\left[\begin{array}{c} E_{z}^{m}\\ H_{y}^{m} \end{array}\right]=\left\{A_{f}^{m}e^{i\frac{2\pi }{\lambda }k_{xm}(x-x_{m})}\left[\begin{array}{c} 1\\ \frac{i\cdot \gamma_{m}\cdot k_{z}-\varepsilon_{dm}\cdot k_{xm}}{\varepsilon_{dm}-k_{z}^{2}} \end{array}\right]\right\}e^{\frac{2\pi }{\lambda }i\left(-c\cdot t+k_{z}z\right)}+c.c.$$

where Im(kxm)>0. From Equation (7) we have

(21) $$Z_{m}=1$$

Since Z_1_ also can be calculated from known value of Z_m_ using Equation (17), the **dispersion relation for the plasmons in multilayer MO slab** will be

(22) F1,2[T2...[Ti[Fi,i+1[...Tm−1[Fm−1,m[1]]]]]]=−1

where

(23) Tj[Z]=Z−i·tan(2πλkxjtj)1−i·Z·tan(2πλkxjtj)Fj,j+1[Z]=[i·γj·kz−(i·γj+1·kz−Z·εd(j+1)·kx(j+1))εdj−kz2εd(j+1)−kz2]/(εdj·kxj)kxj2=εdj−kz2−γj2/εdjIm(kxm)>0Im(kx1)>0

The equivalent form of dispersion relation can be derived by calculating the value of Z_m_ from known value of Z_1_

(24) $$F_{m,m-1}\left[T\prime_{m-1}...\left[T\prime_{i}\left[F_{i,i-1}\left[...T\prime_{2}\left[F_{2,1}\left[-1\right]\right]\right]\right]\right]\right]=1$$

**Example 1.** Plasmons in single-layer-dielectric/metal structure

Using j=1 for the metal ( $\begin{array}{cc} \varepsilon_{d1}=\varepsilon_{metal} & \gamma_{1}=\gamma \end{array}$) and j=2 for the dielectric ($\begin{array}{cc} \varepsilon_{d2}=\varepsilon_{diel} & \gamma_{2}=0 \end{array}$), the dispersion relation (22) will be

(25) $$F_{12}\left(1\right)=\left[i\cdot \gamma \cdot k_{z}-\left(-\varepsilon_{diel}\cdot k_{x,diel}\right)\frac{\varepsilon_{metal}-k_{z}^{2}}{\varepsilon_{diel}-k_{z}^{2}}\right]/\left(\varepsilon_{metal}\cdot k_{x,metal}\right)=-1$$

or

(26) $$\varepsilon_{metal}\cdot k_{x,metal}+i\cdot \gamma \cdot k_{z}+\frac{\varepsilon_{diel}}{k_{x,diel}}\left(\varepsilon_{metal}-k_{z}^{2}\right)=0$$

where $k_{x,metal}=\sqrt{\varepsilon_{metal}-k_{z}^{2}-\gamma^{2}/\varepsilon_{metal}}$, $k_{x,diel}=\sqrt{\varepsilon_{diel}-k_{z}^{2}}$, Im(kx,metal)>0Im(kx,diel)>0 and $\varepsilon_{metal},\gamma$ are the diagonal and off-diagonal elements of permittivity tensor for the metal.

**Example 2.** Plasmons in double-layer-dielectric/metal structure

Using j=1 for the metal ($\begin{array}{cc} \varepsilon_{d1}=\varepsilon_{metal} & \gamma_{1}=\gamma \end{array}$), j=2 for the dielectric N1 ($\begin{array}{cc} \varepsilon_{d2}=\varepsilon_{diel1} & \gamma_{2}=0 \end{array}$), which is in contact with metal, j=3 the dielectric N2 (substrate) ($\begin{array}{cc} \varepsilon_{d3}=\varepsilon_{diel2} & \gamma_{3}=0 \end{array}$) and t as the thickness of dielectric N1, the Eqns. (23) are simplified as

(27) $$\begin{array}{l} F_{2,3}\left(1\right)=\frac{\varepsilon_{diel2}\cdot k_{x,diel1}}{\varepsilon_{diel1}\cdot k_{x,diel2}}\\ K=T_{2}\left(F_{2,3}\left(1\right)\right)=\frac{\frac{\varepsilon_{diel2}\cdot k_{x,diel1}}{\varepsilon_{diel1}\cdot k_{x,diel2}}-i\cdot tan\left(\frac{2\pi }{\lambda }k_{x,diel1}t\right)}{1-i\cdot \frac{\varepsilon_{diel2}\cdot k_{x,diel1}}{\varepsilon_{diel1}\cdot k_{x,diel2}}\cdot tan\left(\frac{2\pi }{\lambda }k_{x,diel1}t\right)}=\frac{\varepsilon_{diel2}\cdot k_{x,diel1}-i\cdot \varepsilon_{diel1}\cdot k_{x,diel2}\cdot tan\left(\frac{2\pi }{\lambda }k_{x,diel1}t\right)}{\varepsilon_{diel1}\cdot k_{x,diel2}-i\cdot \varepsilon_{diel2}\cdot k_{x,diel1}\cdot tan\left(\frac{2\pi }{\lambda }k_{x,diel1}t\right)} \end{array}$$

and the dispersion relation (22) will be

(28) $$F_{1,2}\left[T_{2}\left(F_{2,3}\left(1\right)\right)\right]=\left[i\cdot \gamma \cdot k_{z}+K\cdot \varepsilon_{diel1}\cdot k_{x,diel1}\frac{\varepsilon_{metal}-k_{z}^{2}}{\varepsilon_{diel1}-k_{z}^{2}}\right]/\left(\varepsilon_{metal}\cdot k_{x,metal}\right)=-1$$

or

(29) $$\varepsilon_{metal}\cdot k_{x,metal}+i\cdot \gamma \cdot k_{z}+K\cdot \frac{\varepsilon_{diel1}}{k_{x,diel1}}\cdot \left(\varepsilon_{metal}-k_{z}^{2}\right)=0$$

where $k_{x,metal}=\sqrt{\varepsilon_{metal}-k_{z}^{2}-\gamma^{2}/\varepsilon_{metal}}$, $k_{x,diel1}=\sqrt{\varepsilon_{diel1}-k_{z}^{2}}$, $k_{x,diel2}=\sqrt{\varepsilon_{diel2}-k_{z}^{2}}$, Im(kx,metal)>0, Im(kx,diel2)>0.

The dispersion relation (22) is obviously valid for surface plasmons propagating in the structure without any MO material. **The dispersion relation for the plasmons in multilayer slab without any MO layer** will be

(30) $$F_{1,2}\left[T_{2}...\left[T_{i}\left[F_{i,i+1}\left[...T_{m-1}\left[F_{m-1,m}\left[1\right]\right]\right]\right]\right]\right]=-1$$

where

(31) Tj[Z]=Z−i·tan(2πλkxjtj)1−i·Z·tan(2πλkxjtj)Fj,j+1[Z]=[Z·εd(j+1)·kx(j+1)εdj−kz2εd(j+1)−kz2]/(εdj·kxj)kxj2=εdj−kz2Im(kxm)>0Im(kx1)>0

## 5. Conclusions

The main merit of a plasmonic optical isolator might be an excellent technological compatibility for the integration into PIC and a broad wavelength operational bandwidth. The relatively high optical loss for the surface plasmons in structures, which contains transition metals, can be significantly reduced utilizing the structures with a double-layer dielectric. We have demonstrated that in this structure the MO FoM may increase to above 100%. This is a significant enlargement of MO FoM compared to 2–8% of the MO FoM, which is the most that can be achieved for the structure with single-layer dielectric.
